# Exploring the social dynamics of urban regeneration: A qualitative analysis of community members' experiences

**DOI:** 10.1111/bjso.12578

**Published:** 2022-09-09

**Authors:** Stacey C. Heath, Anna Rabinovich, Manuela Barreto

**Affiliations:** ^1^ School of Psychology and Counselling The Open University Milton Keynes UK; ^2^ Department of Psychology University of Exeter Exeter UK; ^3^ School of Psychology University of Sussex Brighton UK

**Keywords:** cohesion, community engagement, group processes, intergroup relations, social identity, urban regeneration

## Abstract

The present paper explores psychological processes that underpin the success of community change in the context of urban regeneration schemes. We adopt a social identity approach to develop an understanding of the ways in which social identity dynamics may impact upon peoples' experiences of regeneration, and what influence these identity processes have on the creation of new communities. Qualitative interviews, using thematic analysis as an analytic technique, were conducted with community members (*n* = 14) in a recently (2001–2011) regenerated area in the South‐West of England. Three overarching themes were identified: Patterns of identification, willingness to engage, and the notion of regeneration as an event. The research overall highlights the central role of group‐based identity in understanding the processes of regeneration and how this is experienced by different community members. Findings are discussed in relation to the impact regeneration schemes have on community members' sense of collective self, unity, and engagement. The research highlights the pivotal role of social identity processes in delivering successful and sustainable strategies of urban regeneration.

## INTRODUCTION

Urban regeneration, revitalization, or renewal are terms used to describe the processes of land redevelopment in cities that suffer from urban decay. Such schemes aim to address issues of poor health, educational attainment, well‐being, and employment rates, in an effort to raise the socio‐economic status of an area, and individuals who reside within it (Colantonio & Dixon, 2011; Madden, 2014). However, despite the ubiquity of these schemes, urban regeneration strategies are not uniformly successful, often experiencing problems in the sustainability of community engagement (Trueman et al., 2013; Woolrych & Sixsmith, 2013).

One explanation for this inconsistency is the lack in understanding of what mobilizes community engagement within this context. Indeed, policy makers have stated that generating and sustaining effective community engagement and cohesion is a constant challenge (Stafford et al., 2014). This, according to an array of research and project evaluations, is because strategies often focus heavily on short‐term interventions (Hoban & Beresford, 2001) and produce a sense of distrust following a legacy of imposed or failed programmes (Coaffee, 2004).

Despite this, the idea that community engagement is key to the success of regeneration strategies still prevails among policy makers and across academic literature (e.g., see Kingston et al., 2005). Indeed, governmental policies, community change agents, and local authorities all acknowledge that public services are much more effective, and dramatically increase their resources, when they engage with communities directly (Atkinson, 1999; Fung, 2009). At the same time, there has been little empirical research to date that focusses on socio‐psychological processes and group‐based dynamics related to community engagement in urban regeneration context. The present paper aims to address this gap by reporting a qualitative exploration of urban regeneration experience, with a focus on psychological factors linked to cohesion and engagement. Within this context, we define community engagement as a dynamic process that facilitates communication, involvement, and collaborative work between community members and organizations to achieve outcomes at the individual, organizational, or societal levels. Cohesion, on the other hand, is defined by a sense of belonging and togetherness between the individual and their community that promotes a sense of trust and unity around shared community goals and visions.

### Urban regeneration: Policy and context

The conceptualization and evaluation of regeneration schemes is fraught with difficulties. Indeed, there is no one clearly defined definition or strategy implemented across the board, but rather a multitude of strategies and definitions with differing aims and scope (Roberts, 2000). However, given that areas are identified as in need of regeneration based on a variety of key social ‘problems’ such as high unemployment levels, crime rates, and levels of poverty, the notion of community diversification, which involves integration of wealthier residents into areas of decline, is a commonly adopted solution (Butler & Robson, 2001; Galster, 2007; Raco, 2012). This approach is based on the assumption that such integration will create links to employment, reverse stigmas, provide socio‐cultural role models and values for existing residents, and provide a higher‐income base for attracting private investment (Berube, 2005).

Focussing on socio‐cultural role models by introducing middle‐class residents to regenerated areas, with little or no attention to the group‐based dynamics within this context, however, ignores any deep‐rooted problems that may be causing or exacerbating the state of poverty and deprivation, and may undermine cohesion within existing communities (Tallon, 2013). Therefore, there is an urgent need for regeneration strategies to be driven by a framework that incorporates informed and accurate understanding of the impacts of urban regeneration, and integrates knowledge of how communities cooperate to manage—or resist—planned changes within this context (Bailey et al., 2004; Pethia, 2011; see also Huttunen et al., 2022). To achieve this, it is necessary to develop our understanding of group‐based attitudes and behaviours within regeneration context, as well as establish baseline social impacts as benchmarks for future evaluations of regeneration processes. We intend to address these issues by adopting a group‐based approach that aims to understand the drivers of engagement and cohesion between community members within regenerated communities. From a psychological perspective, positive group cohesion is developed through psychological identification with the group. The social identity approach (Tajfel, 1978; Turner, 1982) provides a theoretical framework for understanding how group‐based identification develops and functions.

### The social identity approach

The social identity approach suggests that when individuals identify as a member of a specific group, they become psychologically attached to that group. When this happens, group members become motivated to act in the interest of that (in)group (Hogg, 2006) and tend to positively differentiate themselves from other (out)group members. Furthermore, the impact of social identification is even more pronounced when the (in)group is perceived to be under threat—in these circumstances, low‐identifiers distance themselves from the group (protecting the self), whereas high‐identifiers work harder to emphasize their collective identity (protecting the collective self; see Ellemers et al., 1997; Rabinovich & Morton, 2012; Spears et al., 1997). This tendency can lead to the denigration and discrimination of outgroup members and lay the foundations for inter‐group competition and conflict (e.g., Sherif, 1966). Given that regeneration processes incorporate large‐scale physical and social changes within communities (which could be perceived as group‐related threat), and often integrate new community members, the social identity framework seems strongly relevant to exploring the intra‐ and inter‐group dynamics of regenerated areas.

Below we briefly outline specific areas of research within the social identity framework that may provide a basis for analysing group processes in the regeneration context. These include the rejection identification model (RIM; Branscombe et al., 1999) which focuses on role of group‐based identification in responses to stigma, the social identity model for collective resilience (SIMCR; Drury, 2012) which explores how group members develop a sense of ‘we‐ness’ in challenging environments, and work on the role of group identification in responses to change in organizational and community contexts.

Research conducted within the RIM framework demonstrates that when adversity and stigma are salient, members of stigmatized groups often respond by emphasizing levels of in‐group identification. This increased identification is conceptualized as a coping response to stigma, fostering well‐being, belonging, and life satisfaction, and buffering against the overall costs of exclusion, rejection, and discrimination. These findings have been supported across a variety of stigmatized groups, such as international students (Schmitt et al., 2003), multiracial individuals (Giamo et al., 2012), disabled people (Bogart et al., 2017), and people with body piercings (Jetten et al., 2001). Given that regeneration strategies are identified as necessary based on levels of deprivation and perceived social dysfunction, applying principles of RIM could help bolster in‐group identities that work to protect the well‐being of the existing members of disadvantaged communities.

However, in addition to stigmatized group members reacting to stigma in‐line with RIM principles, there is also evidence of stigmatized members reacting in‐line with a rejection *dis*identification model (RDIM; Jasinskaja‐Lahti et al., 2009)—that is, distancing themselves from the discriminating out‐group. Urban regeneration strategies often incorporate the integration of different sub‐groups (i.e., stigmatized existing community members and middle‐class new owner occupiers), and require these different sub‐groups and community agencies to engage collectively, thus, processes of RDIM may also occur, putting communities at risk of further segregation. These insights highlight the importance of understanding how, and under what circumstances, ‘shared identities’ might develop.

Research conducted within the SIMCR (Drury, 2012) explores how individuals within a group develop a sense of ‘we‐ness’ in challenging environments, suggesting that when people perceive themselves as part of a group in adverse circumstances, several cognitive shifts occur. Specifically, individuals self‐define and identify as members of that group, see other in‐group members as part of the (collective‐)self, develop a common group‐based identity, and adopt group‐normative actions and behaviours. SIMCR argues that this process of switching from ‘me’ to ‘we’ and perceiving oneself in terms of the collective‐self can occur even among strangers when people perceive a sense of common fate—for example, when facing a natural disaster or mass emergency (Drury et al., 2009a, 2009b). This research suggests that developing a shared identity based on perceptions of common fate can act as a resource to motivate co‐operative, group‐focussed behaviours and protect group members against threat and consequences of disaster, providing a basis for an adaptive and collective response by the group (see also Rabinovich et al., 2020). Applying these principles to the context or urban regeneration, SIMCR may be used to understand when and by what means individuals begin to identify as members of a wider, previously (or currently) unfamiliar community under the challenging circumstances of the regeneration process.

However, given that SIMCR has only been successfully applied to newly identified groups as a reaction to external threats, it may not be sufficient for fully understanding how community identification might develop between new *and* existing community members, or what impact this might have on overall engagement with the external agencies who deliver the regeneration process. To further understand the complexity of these inter‐group dynamics and what might motivate individuals to engage with a wider superordinate community as an in‐group, it may be necessary to consider research that addresses group‐based change in community and organizational contexts.

Research that looks at the role of group identification in successfully adapting to community change demonstrates that increased identification with the overarching community leads to increased levels of well‐being, belonging, acceptance, and reduced intergroup anxieties (Cruwys et al., 2022; Heath et al., 2017; Stevenson et al., 2018, 2020). However, in‐line with RDIM principles, research within this domain has also demonstrated stigmatization of disadvantaged communities to undermine relations between service providers and community members (Stevenson et al., 2018). This body of research is particularly relevant when attempting to understand possible reasons for low community engagement with the regeneration strategy itself. Interestingly, when individuals do engage with community organizations, an increased sense of belonging and quality of life has been shown (Haslam et al., 2020), suggesting that social identification may act as a platform to protect against the negative effects of large‐scale community change.

Another body of research relevant to understanding the impact of identity change has been conducted in the context of organizational mergers (Jetten et al., 2002). This research demonstrates that higher identification with a super‐ordinate group relative to a subgroup (i.e., with the organization, relative to a work‐team) fosters more positive feelings about the merger, suggesting that a wider group‐identity could lead to positive change. In addition, in the context of organizational change, research has evidenced the sense of voice perceived by group members as a key strategy to developing identification within a new place or organization, and pivotal to the successful change process (e.g., Knight & Haslam, 2010). Importantly, Farndale et al. (2011) suggest it is the perception of voice, rather than whether voice mechanisms actually exist, that impact upon individuals' level of commitment and identification with the organization.

To summarize, there is solid evidence that group‐based identification is associated with a greater ability to cope with stigma, and may foster a willingness to engage with a superordinate group, and contribute to its goals (even when that group is previously unfamiliar). Applying these insights to the regeneration context suggests that community identification may be a key driver of community engagement with the regeneration projects, leading to a more sustainable positive change.

However, despite the apparent relevance of the research highlighted above, which applies concepts of the social identity approach to stigmatized groups, newly formed groups, and changing communities and organizations, it does not explore the experiences of large‐scale one‐off community change, and the drivers of motivation and engagement in the context of such change. Based on the above research, it could be suggested that identifying with one's community provides residents of regenerated areas with psychological resources that could have positive implications for their well‐being, overall willingness to contribute towards collective goals, and resilience to future change. The present research aims to start exploring this possibility by developing knowledge around residents' experiences of urban regeneration, and qualitatively exploring identity dynamics of a regenerated community, as well as psycho‐social drivers (and barriers) of engagement within this context.

### The present study context

To address the above research question, interviews were conducted in a recently (2001–2011) regenerated area in the South‐West of England. The exact demographics of the area are unknown, however, local council informed us that the area houses around 5000 people, was originally built in the eighteenth century to house workers in the royal navy, and was identified in the 1990's as the most deprived and transient community in the city and in need of regeneration. The UK government awarded a ‘new deal for communities' grant for an extensive regeneration program in the focus area that aimed to “create a thriving and vibrant community that raises aspiration, grasps opportunities and which has people queuing to join” ([Area] regeneration committee, 2001, p. 2). Many residents were moved out of their properties into other available social housing across the South‐West, so that existing housing could be demolished to allow for homogenous new‐builds that “encompass a mix of dwelling types and tenures which encourages social and economic cohesion” ([Area] action plan, 2006, p. 11). After the newbuilds were complete, some of the previous residents had the option to return, whereas others did not. The criteria for this, and number of residents displaced, is unclear, but it was never possible for all residents that were moved out to return. By the time of the interviews, the regeneration programme had finished, and any temporarily displaced community members had re‐settled back into the community, making it an ideal community to explore people's experiences of regeneration, since the scheme could be reflected upon, but was still salient in people's minds.

## METHODS

The analysis was conducted using thematic analysis as a process to assess participants' perceptions and understandings of social relations in the context of regenerated communities. All members of the research team have had experience of working with stigmatized populations across the breadth of their research. Moreover, relating to reflexivity, the interviewer (and the first author) has had direct experience of previously living in, and working with, regenerated communities, allowing for a basic understanding of the regeneration process and experience. Despite this awareness, however, many residents perceived the researcher as council representation: For example, when discussing regeneration during recruitment, some residents responded with comments such as ‘you can tell the council they need to finish ‘x’ and ‘are the council going to re‐open the forum?’. To minimize these perceptions, the interviewer made it clear both verbally and through the information and consent forms that the research forms part of a PhD study that looks to explore the experiences of regeneration, and is in no way tied to the local council.

### Participants

Participants (*N* = 14) were recruited using convenience and snowball sampling between December 2014 and February 2015, in a community café with owners' permission. Sixteen participants were approached in the first instance, however two declined to take part in a formal interview. All participants were white British, aged between 20 and 68 ($\bar{\text{x}}$ = 41; males *n* = 6, $\bar{\text{x}}$ = 42; females *n* = 8, $\bar{\text{x}}$ = 40) and fell into one of four categories (see Table 1): (i) remaining residents who resided in the community pre‐regeneration (*n* = 6), (ii) younger residents who ‘grew up’ through the regeneration process (*n* = 2), (iii) new residents who came to reside in the area during or after the regeneration (*n* = 4), and (iv) working community members who have come to work, but do not live, in the area following the regeneration (*n* = 2). Including these four categories was a deliberate attempt to gain insight into the community from the perspective of different members to identify (where relevant) any inter‐group boundaries that may (or not) be present.

**TABLE 1:** Participant demographics and categories

Interviewee	Age	Gender	Resident category
Interviewee 1	42	Male	i – Pre‐regeneration remaining resident
Interviewee 2	20	Female	ii – Younger resident
Interviewee 3	29	Male	iiii – New post regeneration worker
Interviewee 4	68	Female	i – Pre‐regeneration remaining resident
Interviewee 5	54	Female	iii – New post regeneration resident
Interviewee 6	38	Female	i – Pre‐regeneration remaining resident
Interviewee 7	39	Female	iii – New post regeneration resident
Interviewee 8	54	Male	i – Pre‐regeneration remaining resident
Interviewee 9	39	Male	i – Pre‐regeneration remaining resident
Interviewee 10	32	Male	iiii – New post regeneration worker
Interviewee 11	42	Female	iii – New post regeneration resident
Interviewee 12	36	Female	iii – New post regeneration resident
Interviewee 13	23	Female	ii – Younger resident
Interviewee 14	56	Male	i – Pre‐regeneration remaining resident

Sample size was determined based on reality constraints. Within a hard‐to‐reach community, it is difficult to engage community members, especially as an unknown person who represents an unfamiliar organization. Despite these challenges it was clear after 14 interviews that data saturation was reached, with no new phenomena emerging. This is in line with Guest et al. (2006) who suggest that data saturation can occur within the first 12 interviews, with very few phenomena emerging in any subsequent interviews. The only exception may have been from the perspective of new workers, however, given that these interviewees do not reside in the area, their perspective was included as a guide to external perception only, and two interviews were considered enough to capture this perspective.

### Materials and procedure

Once consent was obtained, participants were interviewed, and audio recorded at the café; each interview lasted between 20 and 55 min. The interview schedule was designed partly exploratory, to understand experiences from the perspective of residents (e.g., ‘What can you tell me about it [regeneration]?’), but also theoretically driven, to explore the inter‐ and intra‐group dynamics of community regeneration (e.g., ‘how do you feel about other members of [the] community?’; see Appendix S1 for full materials). Participation in the study was voluntary with no remuneration offered. The participants were debriefed following each interview.

### Data analysis

The transcriptions were analysed using thematic analysis methodology following the six‐phase procedure (Braun & Clarke, 2006). Since the rationale for this study was to explore experiences of regeneration from the perspective of community residents, and to better understand identity dynamics and the impact on engagement within this context, the research questions were both theoretically driven by the social identity framework, and exploratory. Therefore, a contextualist framework was adopted incorporating both realist and constructionist elements to give both participants and researcher the scope to explore the context of individual interviews outside the parameters of the pre‐designed questions.

The realist method enables participants to express the reality of their experiences (of regeneration), and the meanings attached to these experiences, through their accounts (Braun & Clarke, 2006). This approach is necessary to help the researcher understand the experience of regeneration from the perspective of the residents, while maintaining sensitivity to meanings that do not fit with the chosen theoretical account (i.e., the social identity approach).

Social constructionism, in contrast, views realities as actively constructed within specific social contexts in order to achieve and reflect the meanings we perceive as realities (Taylor & Ussher, 2001). Given that this research is theoretically driven by the social identity approach, incorporating a constructionist framework enabled the researchers to focus on the sociocultural context and structural conditions of the participants' accounts (Braun & Clarke, 2006, 2012).

Within this study, and in line with the contextualist approach, the researcher adopted a combination of inductive and deductive thematic analysis (Miles & Huberman, 1994). On the one hand, the data was coded inductively at a semantic level: We took explicit meanings from the surface of the data with the themes identified being linked to the data itself (Braun & Clarke, 2006), allowing participants to express meaning and experience through language (Potter & Wetherell, 1987). On the other hand, theoretical and ideological constructs of the social identity approach, (such as “Identity change”, for example) were used to deductively identify themes within the data.

Initial codes, identified as analytically relevant to addressing the research questions, were developed from the raw data and supported by the computer package *nvivo11* (2015). A sample of the coded data was discussed and re‐examined with the research team for reliability. Once agreement was achieved, all codes were reviewed to identify areas of similarity and overlap, allowing for the collapsing and clustering of codes into themes (Braun & Clarke, 2012). In addition to those codes that were constructed based on the raw data, theme definitions were also influenced by concepts from the literature.

## RESULTS

The final analysis identified three broad themes: (i) patterns of identification, where the impacts of the physical, social, and demographic changes to the community were discussed in both positive and negative ways; (ii) perceptions of regeneration as a one‐off event, where regeneration was discussed as a one‐off event that has now finished, with all resources withdrawn; and (iii) a willingness to contribute and engage, where contribution was discussed by some as a positive form of engagement, increasing one's sense of connection and inclusion, whereas others discussed contribution as a one‐off event in reaction to a direct need. Each of these themes contain sub‐themes that were created to give structure to the complexity of each broad theme, and to demonstrate hierarchy within the data (see Table 2; Braun & Clarke, 2006).

**TABLE 2:** Themes and sub‐themes

General themes	Sub‐themes	Description
Patterns of Identification: Physical, Social, and Demographic Change	Regeneration improves safety perceptions Division of community, undermining unity Meta‐perceptions—negative image of the community Loss of autonomy and choice Lack of identity clarity Marginalization Stigma Withdrawal of resources	Participants discuss the changes to (the area), and how these impact upon the community's previous and current identity. Participants who were very young when the regeneration scheme started, attach positive meaning to the changes experienced. They believe that these changes allow the old identity to be forgotten, and a new identity can be created which challenges negative external perceptions of the area. Older residents, by contrast, discuss the changes in both positive and negative ways: Changes to the physical environment are perceived positively, while demographic changes to the area are referred to as a “broken community.” This sense of negativity is often discussed with reference to the withdrawal of services and lack of sustainability
Regeneration as an event: The (un)sustainability of imposed change	Withdrawal of services, feelings of abandonment Increases in a sense of community and cohesion Perceptions of dependency Lack of sustainability post ‘event’	Participants talk about the regeneration scheme as an event that happened in the past and has now finished. These perceptions of regeneration as an event that has passed lead to negative discourse around the withdrawal of services and funding that were linked to the regeneration process
Willingness to contribute: The role of identification and remuneration in motivating engagement	Engagement can foster support Contribution as a one‐off event Quid pro quo perceptions of contribution Incentives for engagement Council as outgroup	Willingness to contribute is discussed by participants in a number of different ways. Some see contribution to the community as a positive form of engagement, increasing their sense of connection and inclusion. However, others see the act of contribution as a one‐off event, usually in response to a direct need that arises, such as a block of flats burning down. Furthermore, the regeneration strategy, while in the process of engaging community members, has created a baseline expectation of remuneration for contribution. Finally, some participants, when talking about being actively involved in the regeneration scheme itself, discussed feelings of negativity and a loss of community identity. The volunteers are not being seen by other community members as part of the community but are rather perceived as advocates for the council

### Patterns of identification: Physical, social, and demographic change

The social and demographic changes to the area were discussed in two juxtaposing ways. Remaining participants, who were integrated within the community before the regeneration, referred to the social changes as a division of community. For example, one (remaining) female resident talked about how people “had to move out, they all moved out and stayed out” and how these changes affected the community: “Obviously it…divided it I suppose, because it moved people out” (int 1). Similarly, another remaining community member talked about the regeneration leaving the community “diluted” saying:

Extract 1 Over the years it has been diluted…because people were decanted out…and not everybody came back…and obviously new people got moved in. (int 14)

Other community members discuss this change as a lack of choice, where people were moved from their council^1^ properties to allow new properties to be built for private owners:

Extract 2 She [a friend] lived in the flats that got bulldozed down, and no the tenants did not have…choice cos basically they were told you either move…or you lose your home. (int 11)

This lack of choice was discussed as a result of the social position that inhabitants of social housing estates occupy, with a perceived dependency on resources being used as a (rhetorical) justification for a lack of choice:

Extract 3 Everything just being done to them cos they are in social housing, a lot of them aren't working…you end up with this reliance on the state, on the authority, reliance on your housing association and then regeneration. It is definitely this theory of being done to…you are not being given a voice…it's just being done to you, like you have no choice. (int 3)

These extracts highlight the lack of choice, loss of autonomy and marginalization that surrounded the physical regeneration, from the perspective of new and remaining residents in the community. However, the younger residents who grew‐up during regeneration, discuss the changes to the community more positively, with increases in a sense of safety linked to the demographic changes:

Extract 4 When I was younger you did not feel safe walking down the street by yourself…there were a lot of ex‐convicts, drug dealers, alcohol users, and it was quite scary. But now that's all changed. (Int 2)

These excerpts show how remaining, younger, and new residents of the community can experience the same process of demographic change differently. Despite these contrasting views, however, the participants generally position themselves as community members, and would rather preserve the current identity than develop an entirely new one:

Extract 5 It's not all perfect but you perhaps need to have new faces coming in who know how things work…who have contacts and know how the council works and things like that. But…I do not want the character to change. (int 12)

However, this current identity is also discussed as somewhat unstable, created through the regeneration scheme itself but not having established any firm roots, given the relatively short period of time that the regeneration scheme lasted, and the vast changes imposed onto the community:

Extract 6 It just created this new kind of sense of community, that we were capable of being something but…when you bring that sort of program to a close…things quickly fall back into the channel and you carry on as you were before, before but different, because it was a decade of regeneration, you cannot really remember who you were before, and the services that were brought by the regeneration, and all that the community got involved in with that scheme has gone, so what's left? (int 14)

This participant suggests that the regeneration scheme created a new identity, but now that the project has finished, this new identity has gone, and the previous identity has also been lost, with community members being unable to remember who they were before, highlighting a lack of clarity about who the community are now. This is further discussed by some participants with reference to the withdrawal of services following project completion further dividing the new community:

Extract 7 The regeneration dividing the community and then just leaving ten years later, leaving it divided with new people what we do not know…it just separated what was a very strong single community area into little pockets of communities with their own little focuses. (int 13)

Participants also talk about how the demographic changes that have occurred as a result of the regeneration have undermined the community's sense of unity, developing subgroup identities based on class and status:

Extract 8 The demographics of the area are changing, it's like each row is something different, so where my friend lives she's got all young professionals in her row, I tend to still have more traditional [AREA] families around me, so we do not have too much in common (int 7)

The new and younger community members frequently discussed their community‐based identity by referring to the image that their community may display for external observers, suggesting that the external image is not consistent with the physical and demographic changes that have occurred through the regeneration scheme:

Extract 9 I think it'll just be trying to break that stigma of areas have bad names, and I think that's why people [external to community] still have that issue about us, because they think all about the stigmas more than the people that's around. (int 2)

This sense of identity, of “who we are” as a community, is juxtaposed against outsiders' perceptions:

Extract 10 When I told people I was moving to [Area], my friends were horrified…it used to have a really bad reputation…my friends used to go ‘oh bandit country’ but I've always liked it, and if they could only see because the joke's on them… to me, it's like being back in an old‐fashioned community again. (int 5)

Participants presented the changes to the community as a means to challenge the perceived stigmas attached to the area by the wider city.

Extract 11 When I was young there was this huge stigma about [Area], it wasn't safe, you cannot go there alone, you get robbed or burgled, whatever…but if you spend an hour [here] you can see it's definitely not like everyone's saying…that's definitely not the situation, it's not how we have been portrayed. (int 2)

Other participants acknowledge these negative representations and resist them by positioning themselves as highly identified members of the group:

Extract 12 I just find it a kind place to live. And I say the joke is on the [local media]…it said that this is the crime capital of the west country, and I thought not since I've been here, not the bit that I'm in…in my old place, we used to look out to see whose cars had been vandalised, and that's in posh [area]. (int 5)

Here, the participant rejects the views of the media, showing resentment towards external critics, defining them as a ‘joke’ and suggesting that, actually, it is the ‘posh’ area that is violent and criminal. The participant goes on to re‐affirm her identity: “it's just great here, it's home”.

Overall, when talking about demographic change and identity dynamics within the community, participants express positive views about the regeneration scheme itself, suggesting that it has made the area feel safer and helped improve the external image of the area. However, the interviews also reveal some participants experiencing a sense of loss and division within the community, suggesting that the demographic changes have undermined the previous social dynamics by dividing the community and creating sub‐groups.

### Regeneration as an event: The unsustainability of imposed change

Generally, participants talked about the regeneration as a positive scheme which helped foster a sense of community and cohesion during the time it was conducted:

Extract 13 [The] Park is unbelievable…it used to be derelict, and nobody went there, and now the place is buzzing, you've got families, you've got the bandstand up and running…every Sunday during the warmer weather they'd have a kind of like community thing once a month, it was just wonderful. (int 5)

Participants talk about the events that the regeneration scheme held as bringing the community together, offering a taste of the way ‘[it] can be’:

Extract 14 Towards the end of that program, the community was really close‐knit again, and there were summer events, drew in thousands of people and sort of just one day showcased how amazing [it] can be, cos pretty much all the people that came to those events were local people. (int 13)

These extracts discuss increased community cohesion in reference to the events that were organized by the regeneration scheme. However, this positive discourse often refers to the regeneration scheme as an event that occurred in the past and is now finished, and is consequently linked to negative discourse and feelings of regret about the regeneration process ending, and positive changes now being in the past:

Extract 15 It's such a shame that they did not have more years at [area] regeneration…it was brilliant then and it did make a fantastic change. (int 4)

Additionally, the regeneration ‘event’ was also suggested to create an external perception of dependency on local authority that could not be sustained:

Extract 16 If you just give a community a load of money to spend, it will spend it, but if you do not give it a way of making it sustainable or be thinking about the future…there is always this, look we have just got to spend it, and I think, if you do that over a sustained period of time, the community gets er, used to those events. (int 10)

This creation of an over‐reliant community is perceived to create further problems post regeneration due to the dependency and lack of sustainability of delivering short‐term solutions to long‐term problems:

Extract 17 There's also an element that you actually kind of start providing services and knowledge and expertise during the term of the program which you cannot sustain beyond it…delivering that additional resource which will never be sustainable, doing these quick wins. (int 14)

Furthermore, the withdrawal of services following the completion of the regeneration scheme, and the lack of post‐regeneration dialogue, created a perception that the whole regeneration project could be serving the council's objectives rather than the community itself:

Extract 18 But as soon as that final signature was dry…the final bit of regeneration was signed off, no need for that group [neighbourhood forum] anymore, despite the community saying there is because we still want to be able to feed into things…but it does not matter, it's done, not needed to tick their local authority box of engagement so, therefore, not resourced anymore. (int 3)

Some participants also talked about a sense of abandonment by the council that leads to a perceived loss of efficacy and pessimistic expectations for the future:

Extract 19 I think it will go downhill now, like before…because we're not in it together no more, I mean before, it was brilliant…But then of course all that's gone now, it's like it was a bit of a show to say we've done somet[hing] here…it's just a shame that all this good work and money and we're left to dwindle and fall flat again. (int 6)

Here the participant describes the investment as a ‘show’, and states that without support the community cannot survive as it is, implying it will return to the pre‐regeneration state. This dependency on further investment is discussed by some participants in relation to the lack of agency and sustainable support:

Extract 20 You raise people's aspirations to say yeah we can do all this but then they became reliant on somebody else delivering it, and then it just goes, and they do not feel like they can do it for themselves, cos they have always had the help. (int 8)

Following on from this perception of abandonment, some participants suggest that more help is needed for the community to become successful and sustainable, highlighting a need for resources to be directed at social, rather than purely physical, changes:

Extract 21 Building that sense of community will not happen through physical regeneration, that's that sort of more social catching up, but of course there is not such thing as a social regeneration but that's what's needed now I guess, that's the best way of describing it. (int 3)

Overall, participant's position the regeneration as an event that was delivered by local authorities that has now finished. While the regeneration was taking place, participants perceived it as something positive that helped develop a sense of community cohesion and bring community members together. However, subsequent withdrawal of resources, coupled with the delivery of relatively short‐term fixes to long‐term problems, created a sense of dependency on local authority, which is difficult to sustain post‐regeneration. This leaves the community with a lack of direction which, according to some participants, represents a risk of the community returning to its original pre‐regeneration state.

### Willingness to contribute: The role of identification and remuneration in motivating

#### Engagement

Contributions to the community were discussed by participants in a number of different ways. When participants talk about contributing to the community directly or having another resident help them with small‐scale, day‐to‐day activities, contribution is discussed positively:

Extract 22 Regular litter picks and things like that…I like to get involved in small‐scale things like that, not pointing the finger, but just getting people involved. (int 7)

Some members discussed contribution in terms of inclusion and support, knowing that help is there, if needed:

Extract 23 Your close friends you know in the community, your neighbours and friends will, if you need a little bit of help, they'll help you, you can go to them and they'll help you. (int 9)

However, similar to the regeneration itself, contributions were also discussed as one‐off instances. In the extract below a participant refers to a specific event and juxtaposes this type of contribution against a continuous willingness to contribute:

Extract 24 Points of crisis bring loads of people running to help but then, as soon as that crisis has died down, there is not the same, I do not really wanna use the word glamour but there is not that same sort of desire to be part of that first wave of people helping…that quickly dies off. (int 10)

Participants also discussed engagement in terms of rewards, suggesting that the regeneration scheme changed the way community members perceive engagement by creating an expectation of remuneration for contribution because of the incentives offered by council for community engagement:

Extract 25 Councils are always looking to engage those who do not, so they offer an incentive which creates this baseline expectation. (int 3)

This expectation is suggested to affect one's willingness to contribute to the community, where contribution is now influenced by how much people expect to get in return:

Extract 26 There's a historic issue around community engagement in [area], certainly for the last 15 years during the funding…spending money became part of the nature of the area with a what's in it for me…expectation that you are gonna get something for getting involved. (int 14)

Furthermore, when talking directly about contribution to the community through engagement with the regeneration scheme itself, participants describe negative experiences that affect contributors' sense of connection and esteem:

Extract 27 If you are a member of the community and you step up and want to be able to have more responsibility, more of an involvement in change, you kind of lose your connection with your own community. (int 1)

Extract 28 I think you've got to be very lucky to get a councillor who's really still perceived to be a member of the community. I think as soon as you step up to a board level or take some responsibility for something, you're perceived as something completely different. (int 13)

Here participants discuss the impact on community volunteers who become associated with the council, and how this association changes perception of these community members. Participants highlighted that those community members who volunteered during the regeneration initiative were seen as advocates for the council, and their motives were questioned:

Extract 29 People got scared off because they thought why should I get involved with stuff when I'm being put under the spotlight, being attacked for just being part of something I want to improve, you know, my neighbourhood, my community, my local area, so I saw that and it was kind of heart‐breaking. (int 13)

Overall, participants suggest that the regeneration scheme had an impact on residents' willingness to contribute to the community. For some, volunteering to represent the community in the regeneration engagement plan meant they became negatively associated with local authority and were treated less like community members. Moreover, some participants suggest that the regeneration scheme created a ‘quid pro‐quo’ culture, where one's willingness to contribute became dependent on the gain that could be received in return.

## DISCUSSION

This study aimed to develop our understanding of residents' experiences of regeneration and the related social identity dynamics. The research draws on the social identity framework (Tajfel & Turner, 1979) to understand the role of identity processes in intra‐ and inter‐group dynamics and community engagement following large‐scale community change (i.e., urban regeneration).

First, the data highlights some intergroup identity dynamics that arise through the process of regeneration, where in‐group versus out‐group categorizations structure and give meaning to participants' experiences. For example, consistent with the aforementioned RIM principles, some newer residents talked positively about the community (in‐group), positioning themselves as high identifiers and actively constructing ‘posh’ areas as out‐groups, attempting to discredit their opinion of the area (extract 12). Interestingly, these community residents also highlighted some *intra*‐group dynamics distancing themselves from *some* community members and stating that they do not have much in common with certain *traditional* residents (extract 8).

Another example of identity dynamics, and in line with the above‐mentioned.

RDIM principles, were highlighted by some participants discussing their perception of regeneration agencies (such as the council) as an out‐group (extracts 27, 28, and 29). In line with the research by Stevenson et al. (2018), actively defining service providers (such as regeneration agencies and the council) as out‐groups highlights the inter‐group divide between community and the agencies. Such intergroup dynamics could lead to some community members disengaging from both community services and the wider community.

Furthermore, the data suggests that beyond perception of community services as an out‐group, these out‐group perceptions are also extended to community members who engage with the regeneration agencies. This suggests that in‐group members who break norms (of non‐cooperation) often face particularly harsh derogation by other in‐group members (extracts 27, 28, & 29). Such intergroup dynamics could be one explanation of why community engagement in regeneration strategies is generally low, but also might explain why engagement often decreases throughout the course of regeneration programmes.

Another aspect of identity dynamics is the different impacts regeneration has on residents' perceptions of community identity depending on their age and background. In particular, the data suggests there is a divide between remaining residents who have lost their (previous) community identity, younger residents, who were too young to have established a sense of (community‐based) identity pre‐regeneration, and new residents who have come to live in the area post‐regeneration and have created a new identity.

Given that the regeneration scheme was running for 10 years, it is possible that younger residents identify more strongly with the regeneration scheme itself as part of their sense of community, which might be the premise behind the positive perception of the regeneration by younger people. In line with the previously mentioned research on organizational change (Jetten et al., 2002; see also Knight & Haslam, 2010) this difference in perception between younger and older residents could create a barrier to development of a unified sense of community identification, in so far as younger residents are likely to ‘merge’ more easily with new residents compared with older ones.

Despite the sense of loss highlighted by remaining community residents, new and younger residents perceived the overall development of a new regenerated community identity positively, positioning external negative perceptions of the area as a ‘*joke*’, with community residents (in‐group) being described as *‘decent people’*. This is in line with the social identity theory which suggests that when individuals identify with a group, they are motivated to boost their self‐image through the positive evaluation of that in‐group (compared with an out‐group; Tajfel & Turner, 1979). Accordingly, some participants attempt to challenge negative external perceptions of the area and highlight the area's positive attributes by explaining what the area is not (extracts 10 and 11). This is consistent with the theory‐based expectation that positive in‐group distinctiveness can be maintained through intergroup comparisons (Packer & Van Bavel, 2014).

Nevertheless, achieving positive group distinctiveness is not always easy, and this is especially true for members of low‐status groups (Jetten et al., 2012). One reason for this is social stigma that is often associated with such groups. In the present study, some participants talked about the negative stigmas attached to their community (extracts 9 & 11), and the importance of overcoming these stigmas and creating a more positive group‐based identity.

However, some participants' comments suggest a lack of group‐based identity clarity, discussing past identities (both pre‐regeneration community‐identity and the identity that started developing during the regeneration process) in terms of a loss, with community members no longer remembering ‘*who you were before’* (extract 6). Within the regeneration context, and in line with wider research (De Cremer & Sedikides, 2005), it is suggested that a lack of self‐concept clarity could have detrimental effects on the community's ability to create a positive group‐based identity post‐regeneration, leaving participants feeling as though the area will deteriorate to a pre‐regeneration state (extract 19).

More optimistically, the data also suggests several positive effects of the regeneration scheme. In line with principles outlined in SIMCR, participants talked positively about the regeneration programme, describing frequent identity‐related behaviours during the regeneration, such as attending and helping at various events organized through the programme (extract 14). These events are described as bringing members of the community ‘together’ (extract 14), helping to forge a sense of connection during times of change.

Consistent with previous research (Drury et al., 2015), these findings suggest that shared social identity is a key factor in group members' willingness to offer social support and motivate cooperative group‐focussed behaviours that may serve as a buffer against the impact of imposed change. Furthermore, our data suggests that social support and levels of community engagement were perceived to be higher during the regeneration programme, which may be linked to the higher levels of community identification that seemed to be evident at that time (compared with post‐regeneration).

Based on the present findings, it could be suggested that regeneration schemes may become more successful if they incorporate post‐regeneration identity‐building techniques to help foster and maintain positive community‐based identities that address a lack of self‐concept clarity and are aligned with wider community goals and aspirations to ensure sustainability post‐regeneration.

### Limitations and future research

The present study is an important first step towards understanding community members' experiences of regeneration strategies. The qualitative approach enabled us to gain an in‐depth insight into some of these experiences from the residents' perspective; however, some limitations of the chosen approach need noting. First, despite actively acknowledging our own, theoretically driven, biases by attempting to allow participants to shape our understanding of their experiences, the potential impact of the researchers and their subjectivity needs to be acknowledged (Bryman & Burgess, 2002).

Second, this research only offers the perspective of one community in one area of the UK. It is, therefore, difficult to generalize the findings to other areas of regeneration. One way to overcome this is to conduct similar research in other areas to identify overlaps that could be attributed specifically to the urban regeneration context.

Another limitation is linked to sample homogeneity and participant self‐selection. Recruitment and interviews were conducted with white British participants in a (not‐for‐profit) community café, which, by its very nature, may exclude certain members of the community. The implication of this limitation is that, while the research offers an insight into some aspects of responses to regeneration strategies, it may not reflect experiences of some of the more disengaged community members (which is important, given that disengagement may be a consequence of the regeneration scheme itself).

Finally, the interviews were conducted at one time point, following a decade of regeneration. It is likely that community experiences post‐regeneration may have impacted the current group‐based dynamics, making it difficult to retrospectively highlight perceptions and understandings of regeneration, or make inferences about current group dynamics being a consequence of the regeneration itself. To overcome this, longitudinal research could be conducted during and after regeneration processes to understand the impact of urban regeneration on group‐based dynamics across time points. Such longitudinal studies could be complimented with research in a similar regenerated community that engages with identity‐building techniques (for example see Groups4Health, Haslam & Reicher, 2006; and ASPIRe, Haslam et al., 2003) to enable a clearer empirical understanding of the role of identity in fostering engagement and facilitating sustainable community change within this context.

## CONCLUSION

The present research offers an insight into experiences of urban regeneration and psychological processes involved, highlighting potentially problematic aspects of regeneration strategies, and the impact that this could have on communities. It is suggested that group‐based identification is central to levels of engagement from the outset, and that the abrupt end of regeneration strategies may serve to undermine levels of identification within the community. One possible way to enhance regeneration success could be to incorporate training strategies that extend beyond the boundaries of a time‐limited regeneration project and focus specifically on identity‐building techniques to help develop and maintain a sense of connection between community members, old and new.

## AUTHOR CONTRIBUTIONS

**Anna Rabinovich:** Conceptualization; formal analysis; supervision. **Manuela Barreto:** Supervision.

## CONFLICT OF INTEREST

All authors declare no conflict of interest.

## Supporting information


Appendix S1

## Data Availability

The research data supporting this publication are available as supplementary information accompanying this publication.

## Footnotes

^1^
Council properties is the term given to social housing where individuals can rent properties from local authorities at a reasonable price.
