# Supplementary material for: Exploring the social dynamics of urban regeneration: A qualitative analysis of community members' experiences
Source: Br J Soc Psychol. 2022 Sep 9;62(1):521–39. doi: 10.1111/bjso.12578 (PMC10087222; doi:10.1111/bjso.12578)
Supplement: Supplementary file 1 — Appendix S1 [file BJSO-62-521-s001.pdf]

Appendix 1: Overarching Research Questions and Interview Schedule

• What are community members' experiences of regeneration?

• Why do some people feel, and some do not feel, a sense of connection with their community?

• Why do some people identify, and some do not identify, with others in the community?

• What changes resulting from the regeneration are most salient to participants and what meanings are attached to those changes?

• Why do some people engage with regeneration strategies within their community and some do not? • What barriers do they perceive?

• What are the identity dynamics involved in the regeneration process?

• What are the links between the regeneration process and a sense of connection within the community?

Interview Schedule

Hello, my name is Stacey, I am a PhD student at the university of Exeter and I am asking people from 'Devonport' a few questions about what it's like to live in the area. As a single mother who has come from an area that has undergone regeneration in the past, I am interested in finding out how people within similar communities have experienced these changes. How you feel about your community, what changes you have experienced and whether people generally feel that these changes are better or worse. The information I collect today will help me with a research project I am doing about community changes for my studies.

Background

Q1) Tell me a bit about yourself … How would you describe yourself? What do you like to do in your spare time? Are you a part of any clubs or activities?

Q2) How long have you lived in Devonport?

Q3) Why did you move here, tell me a bit about your

Q4) How do you feel about Devonport as a place to live?

Connection/ Identity

Q5) Is there anybody in Devonport that you feel a close connection to?

Q6) Would you move away if you had the choice?

Q7) Why? – (Fit)

Could you change this?

What would need to happen to change this?

Q8) How do you feel about the other members of Devonport community?

Q9) Do you feel that you have much in common with them?

Q10) Why?

Q11) How do people in Devonport help each other out?

Engagement, Participation and Renewal Identity

Since 2006, Devonport has undergone some major regeneration work. Prior to the start of these works it is suggested that the condition of the area, the housing and streets, were poor. Since then the area has seen new housing, old flats demolished and streets and open spaces improved.

I would like to find out how (if at all) you feel these changes affect you, and whether these effects are, in your opinion, good or bad.

Q12) Were you here when the changes to the community started?

Q13) What can you tell me about it?

Q14) Did you have any say in the changes that were going to happen?

Q15) Can you tell me about this?

Q16) Were you given the choice to get involved in the changes that occurred?

Q17) Did you join in or get involved in any way?

Q18) Why?

How did that make you feel?

Changes

Q19) What parts of the community have changed?

Q21) How do you think this has affected the community?

Q22) How do you think these changes have affected you?

Q23) Do you feel these changes are for the better or worse?

Q24) Why?

Q25) Was the change needed in the first place?

Q26) Why?

Q27) Do you think more change is needed?

Q28) In what way? / Why not?

Q29) What sort of responses to these changes have you heard?

(prompt) Community members Council Members Regeneration teams Other People who know the area or visit the area?

Q30) What do you think Devonport will be like in the next 5 years?

Q31) Do you think it's possible to improve Devonport?

Q32) How?

Q33) Do you think that there are some things that will never change?

Q34) Why?

Q35) If there were future plans to further change Devonport would you get involved?

Q36) Why? Ideal communities

Q37) What does ‘community’ mean to you?

Q38) If you could pick your perfect community to live in, what would it be like?

Q39) How would this ideal community affect you?

How will it change your life?
